# Covid-19 in South America: clinical and epidemiological characteristics among 381 patients during the early phase of the pandemic in Santiago, Chile

**DOI:** 10.1186/s12879-020-05665-5

**Published:** 2020-12-14

**Authors:** Macarena R. Vial, Anne Peters, Inia Pérez, María Spencer-Sandino, Mario Barbé, Lorena Porte, Thomas Weitzel, Mabel Aylwin, Pablo Vial, Rafael Araos, Jose M. Munita, for the CAS-UDD Covid-19 Working Group, Alejandra Marcotti, Jorge Pérez, Luis Miguel Noriega, Pablo Gaete, Sebastián Solar, Silvina López, Paulette Legarraga, Valeska Vollrath, Alicia Anderson, Mirentxu Iruretagoyena, Jerónimo Graf, Rodrigo Pérez, Manuela A. Roa

**Affiliations:** 1 Facultad de Medicina Clínica Alemana, Universidad del Desarrollo (CAS-UDD), Santiago, Chile; 2 Present Address: Facultad de Medicina CAS-UDD, Instituto de Ciencias e Innovación en Medicina (ICIM), Santiago, Chile; 3 Millennium Initiative for Collaborative Research On Bacterial Resistance (MICROB-R), Santiago, Chile

**Keywords:** COVID − 19, Coronavirus, SARS-CoV-2, Pneumonia, Epidemiology, South America

## Abstract

**Background:**

Understanding the characteristics of the Covid-19 pandemic in different geographical regions, ethnic and socioeconomic settings are of emerging importance. This study presents the demographic and clinical features of SARS-CoV-2 infected patients in a large private healthcare center in Santiago, Chile, during the first month of the pandemic.

**Methods:**

We analyzed the demographics, laboratory and clinical characteristics including severity and outcome of all patients diagnosed with Covid-19 during the first month of the pandemic. SARS-2-CoV infection was confirmed by RT-PCR in nosopharyngeal samples. The primary outcome was a composite of ICU admission or all-cause, in-hospital mortality. Clinical and laboratory parameters of hospitalized patients were analyzed regarding their association with the primary outcome.

**Results:**

From March 3 to April 4, 2020, 3679 individuals were tested for SARS-CoV-2 in our hospital. Of those, 381 had Covid-19 and were included into this analysis. Most patients (99.2%) were Chileans, 12% returning from recent travel. The median age was 39 years (IQR 31–49) and 52% were female. A total of 88 patients (23.1%) were hospitalized; 18 (3.7%) required ICU and/or died. The overall mortality was 0.7%. Increased body mass index (BMI) and elevated C-reactive protein (CRP) were independently associated with ICU care or death.

**Conclusion:**

During the first weeks of the pandemic in Chile, most Covid-19 patients were young, with low rates of hospitalization, ICU requirement, and fatality. BMI and CRP on admission were predictors for severity. Our data provide important information on the clinical course and outcome of Covid-19 in a Latin American setting.

## Background

In December 2019, a cluster of severe pneumonia of unknown etiology was reported in Wuhan, Province of Hubei, China [1]. A rapid investigation determined that the agent involved was a novel Coronavirus sharing significant sequence identity with bats and human-related coronaviruses [2, 3]. The virus and its associated disease were named Severe Acute Respiratory Syndrome Coronavirus 2 (SARS-CoV-2) and Coronavirus disease 19 (Covid-19), respectively [4]. Soon after its identification, SARS-CoV-2 spread globally causing tremendous burden to health care systems and society as a whole. According to publicly available information, the first case of Covid-19 in Chile was diagnosed on March 3, 2020. Initial cases were imported from Europe, but family clusters and evidence of secondary transmission were rapidly observed. Therefore, the Chilean government declared phase 4 (widespread, ongoing local transmission) on March 16, 2020. Data from early clinical series have shed light on the clinical presentation of Covid-19. After a median incubation time of 5 days [ 5, 6], patients developing symptoms usually present with an influenza-like illness, with fever, dry cough, headache, odynophagia, and dyspnea as the most common symptoms [1]. Interestingly, a large proportion of subjects also report the development of anosmia and/or ageusia as one of the cardinal symptoms of the infection. Early work from China showed that 80% of the subjects with Covid-19 followed an uncomplicated course, 15% required hospital admission and 5% developed a severe infection with a catastrophic respiratory failure needing critical care support [5]. The case-fatality rate is variable, ranging from 2.25% in the Republic of Korea to 14.5% in countries like Italy, with increasing age, and comorbidities being the most important predictors of mortality [7, 8]. As Covid-19 spreads, and the number of cases dramatically increases, a detailed description of Covid-19 in new geographical areas is critical. Moreover, understanding the clinical course and outcomes of patients with Covid-19 in underrepresented populations like Latin America is paramount. In this study, we report the clinical characteristics of Covid-19 in Chile with a focus on subjects hospitalized during the first month of the epidemic.

## Methods

The study was conducted in Clínica Alemana de Santiago (CAS), a tertiary care not-for-profit hospital in Santiago, Chile. CAS is a 442-beds healthcare facility that before the Covid-19 pandemic included 12 general and 10 cardiac intensive care unit (ICU) beds. As a private healthcare institution within Chile’s two-tier health system it mainly serves the higher-income section of the population.

All patients attending the Emergency Room and diagnosed with Covid-19 between March 3 and April 4, 2020, were included in this cohort. Laboratory testing for SARS-CoV-2 infection was performed in the hospital’s molecular laboratory using a commercial RT-PCR kit from nasopharyngeal and oropharyngeal swab samples [9]. Cases were identified using laboratory databases and electronic medical records. Only patients with a positive SARS-CoV-2 RT-PCR were included. Data was extracted and entered into a REDCap database by a team of researchers, after a training session led by the senior data manager investigator (AP). Two study investigators (MS and AP) conducted weekly data audits to ensure the quality of the collected data. Data collected included demographic information, comorbidities, clinical presentation, as well as duration of symptoms, treatment, and outcomes. Laboratory values were automatically extracted from the electronic medical records. The Charlson Comorbidity Index (CCI) was used to summarize comorbidities. The score goes from 0 to 24, with zero representing no comorbidities [10]. Body Mass Index (BMI) was used to assess excess body weight, this data is usually registered at the time of admission, using self-reported data, or after patient evaluation when the information is uncertain. Pulse oximetry saturation (SpO_2_)/ Fraction of inspired oxygen (FiO_2_) ratio was used to assess respiratory exchange. These parameters were assessed regarding their association with the main outcome; a composite of ICU admission or all-cause, in-hospital mortality. Variables used to analyze these risk factors were obtained within 24 h of admission.

### Statistical analysis

Continuous and categorical variables were presented as median (IQR) and n (%), respectively. Patient characteristics were compared by subgroups of interest using a chi-square test, Fisher’s exact test or the Mann-Whitney U test as appropriate. A two-tailed *p*-value < 0.05 was considered statistically significant for all analyses.

To explore risk factors associated with disease severity, univariable and multivariable logistic regression models were used. We selected variables for the logistic regression model based on previous findings and plausibility. A maximum of 8 variables were included in the logistic regression model to avoid overfitting. All analyses were conducted using Stata (Version 16.0. College Station, TX: StataCorp LLC).

## Results

During the first month of the Covid-19 pandemic, 3679 subjects presented with possible SARS-CoV-2 infection and tested by RT-PCR in our institution; 381 (10.4%) were confirmed as positive and included for this study. The average time of symptoms at the time of initial testing was 3.7 days (SD 3.8).

The median age of the cohort was 39 years (IQR 31–49) and 153 (52%) were female (Table 1). A total of 253 (66.4%) patients reported an epidemiological risk factor for acquiring SARS-CoV-2, i.e. 206 had exposure to a confirmed case, and 47 had recently visited a high-risk country, (16 reported both risk factors). Cough and fatigue were the most common symptoms at presentation. A summary of the main demographic and clinical features of the cohort is shown in Table 1.

**Table 1:** Demographic and clinical presentation of patients with Covid-19 diagnosed during the first month of the SARS-CoV-2 pandemic

	All outpatients ***N*** = 293	All inpatients ***N*** = 88	***p***-value
**Characteristic**			
Age, years	37 (28–45)	49 (39.5–65)	<.001
Gender, female	153 (52.2)	45 (51.1)	.85
**Comorbidities**			
Diabetes	5 (1.7)	6 (6.8)	.73
Hypertension	15 (5.1)	25 (28.4)	<.001
BMI ε 30	3 (1)	14 (15.9)	.002
Current smoker	7 (2.4)	6 (6.8)	.055
Obstructive pulmonary disease	10 (3.4)	6 (6.8)	.17
Malignancy	3 (1)	3 (3.4)	.14
Mean CCS (SD)	0.28 (0.7)	1.38 (2.0)	<.001
**Symptoms at the time of initial presentation**			
Cough	170 (58)	64 (72.7)	.013
Fever	167 (43.8)	69 (78.4)	<.001
Odynophagia	126 (43)	35 (39.8)	.59
Fatigue	91 (31.1)	38 (43.2)	.036
Anosmia or Ageusia	28 (9.6)	7 (8)	.65
Chest pain	9 (2.4)	5 (5.7)	.26
Dyspnea	3 (1.0)	4 (4.5)	.048

Data are median (IQR), *n* (%), or n/N (%). *p* values were calculated by Mann-Whitney U test, χ^2^ test, or Fisher’s exact test, as appropriate. χ^2^ test comparing all subcategories

### Subgroup of patients requiring hospitalization

**Fig. 1:**
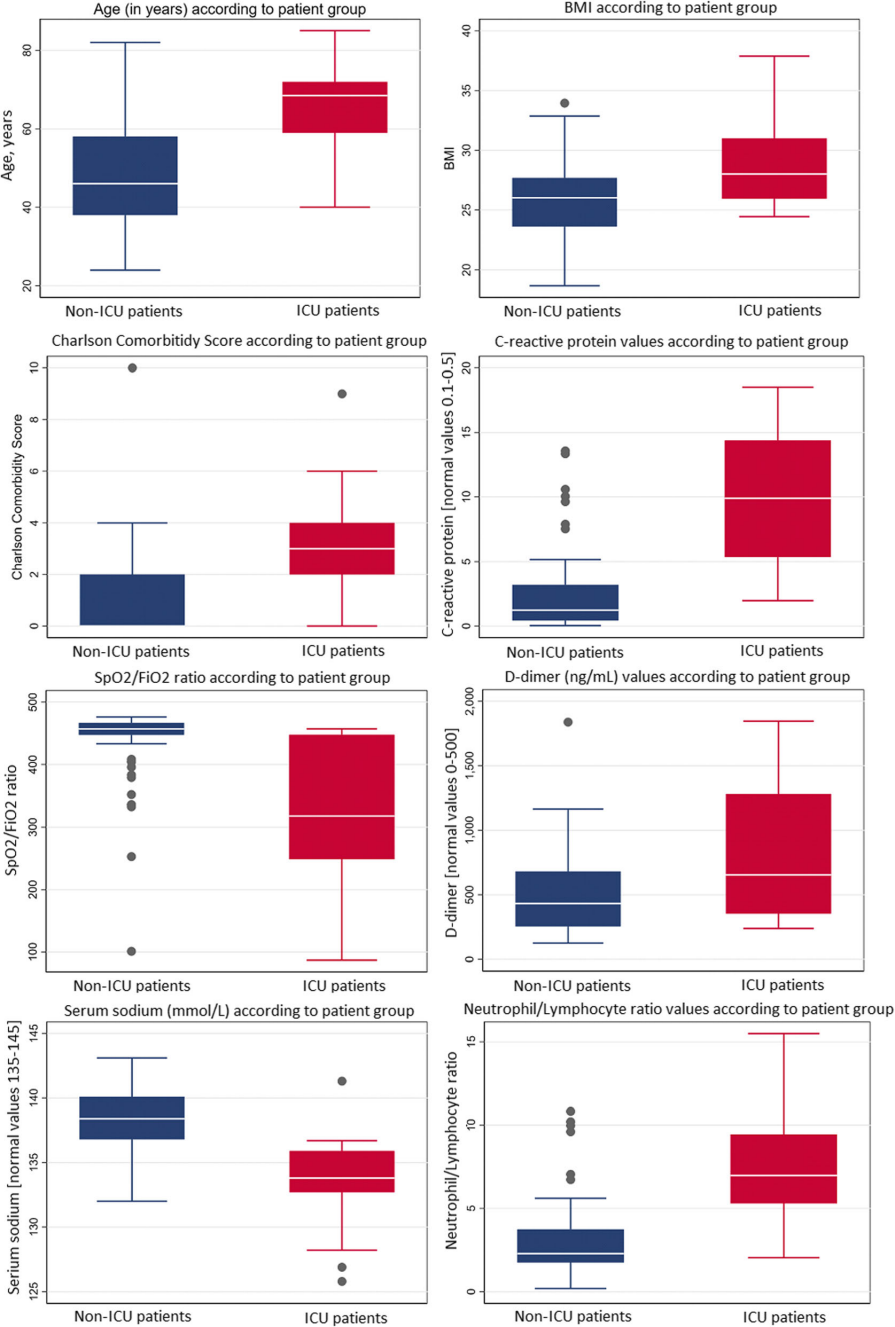
Clinical and laboratory markers within 24 h of admission comparing ICU vs non-ICU patients

Among the 381 patients, 88 (23.1%) eventually required hospital admission, 51 of them were hospitalized after an initial management as outpatients. The median time of symptoms at the time of admission was 8 days (IQR 5–10)*.* Hospitalized patients were generally older than those managed as outpatients (median 49 vs. 37, *p* < 0.001) and had a higher CCS with 0.28 for outpatients and 1.38 for inpatients (p < 0.001). On the first day of hospitalization, 40 (45%) subjects were admitted to general wards, 41 (47%) to a step-down unit, and 7 (8%) to ICU (Table 2). Among subjects hospitalized in general wards and stepdown units, 10 (12%) patients were subsequently transferred to the ICU. In addition, a urinary antigen test for pneumococci or Legionella spp. was ordered in 39 and 41, respectively, with only one of the pneumococcal tests resulting positive. No other patient was found to have a bacterial coinfection at the moment of the admission. The median duration of hospitalization was 8 (4–14.5) days and the median length of stay (LoS) in the ICU was 13 (5.3–17.8) days. A total of 82 (93%) patients had an imaging compatible with pneumonia; 39 (44%) required supplementary O_2_ only, 20 (23%) non-invasive ventilation and 10 (11%) invasive ventilation. Median days of invasive ventilation were 7.5 (6.3–15.3). Among those hospitalized, excess body weight (62.5%) and hypertension (28%) were the most common coexisting conditions. A summary of the laboratory abnormalities on admission is presented in Table 3. The most common laboratory abnormalities were: lymphopenia, increased levels of C-reactive protein (CRP) and D-dimer. Ferritin values were available for 25 patients at admission, with 20 (80%) presenting an increased level. A comparison of patients who presented severe disease, defined as need for ICU care at any time during admission or in-hospital death, with non-severe disease is provided in Table 3. In univariate analysis, odds of ICU care/death were higher in males, and those with higher BMI, older age, a history of diabetes or hypertension, and chronic medication such as steroids, angiotensin-converting enzyme (ACE) inhibitors or angiotensin II receptor blockers (ARBs). Increased white blood cells, neutrophils, CRP, procalcitonin, ferritin, D-dimer, bilirubin, troponin T, LDH and lower SpO2/FiO_2_, prothrombin time, sodium, albumin and lymphocytes were all associated with the need for ICU care or death. Figure 1 is a graphic representation of some of these differences in patients with severe disease (ICU or in hospital death) compared to non-ICU patients. Based on clinical plausibility and significance level on the univariate analysis, the following variables were included in the multivariate logistic regression model: age, gender, CRP, BMI, neutrophil lymphocyte ratio, SpO2/FiO_2_, D-dimer and sodium. Increased BMI and CRP levels were independently associated with increased odds of ICU care/death. SpO2/FiO_2_ had a *p*-value of 0.051, not significant according to our predetermined level of 0.05. See Table 4. As of July 31, out of the 88 patients who required hospitalization, a total of 3 patients had died and 85 had been discharged and remained alive.

**Table 2:** Baseline characteristics at admission of patients who required ICU care or died and those who did not

	All hospitalized ***N*** = 88	ICU care or death ***N*** = 18	Non-ICU care ***N*** = 70	***p***-value
**Demographics**				
Age, years	49 (39.5–65)	68.5 (59–72)	46 (38–58)	.000
Gender, female	45 (51.1)	3 (16.7)	42 (60)	.003
**Symptoms on admission**				
Cough	64 (72.7)	13 (72.2)	51 (72.9)	.96
Fever	69 (78.4)	15 (83.3)	54 (77.1)	.64
Anosmia	7 (8)	0	7 (10)	.16
Ageusia	3 (3.4)	0	3 (4.3)	.37
Odynophagia	35 (39.8)	7 (38.9)	28 (40)	.93
Fatigue	38 (43.2)	6 (33.3)	32 (45.7)	.35
Dyspnea	4 (4.5)	2 (11.1)	2 (2.9)	.16
Chest pain	5 (5.7)	0	5 (7.1)	.24
**Comorbidities**				
Diabetes	6 (6.8)	3 (16.7)	3 (4.3)	.06
Hypertension	25 (28.4)	10 (55.6)	15 (21.4)	.004
Mean BMI (SD)	26.5 (3.8)	29.2 (3.9)	25.7 (3.5)	.002
Smoker	6 (6.8)	1 (5.6)	5 (7.1)	.81
COPD or Asthma	6 (6.8)	3 (16.7)	3 (4.3)	0.097
Mean CCS (SD)	1.38(1.96)	3(2.3)	0.96 (1.62)	.001
**Physical exam**				
Heart rate	81 (74–89)	86 (75–97)	80.5(74–88)	.23
T > 37.5 C (axillary)	42/48 (87.5)	8/9 (88.9)	34/39 (87.2)	0.89
Respiratory rate	20 (18–22)	21(20–24)	20(18–22)	.13
SBP < 90 Or MAP < 65 mmHg	9/81 (11.1)	1/13 (7.7)	8/68 (11.8)	1.0
S SpO2/FiO_2_	452.4 (404.2–461.9)	317.9 (248.6–447.6)	457.14 (447.6–466.7)	<.0001
**Chronic Medications**				
ARBs/ACEi	18/87 (20.7)	9/17 (52.9)	9/70 (12.9)	<.001
Steroids	4 (4.5)	4 (22.2)	0	.001

**Table 3:** Laboratory findings within 24 h of admission comparing patients who required ICU care or died to those who did not require ICU

	All patients	ICU care or death	No ICU care	*p*-value
Hemoglobin, g/dL	14.1 (13.1–14.8)	10.2(10.1–12.5)	14.3 (13.5–15)	.13
White blood cell count, mm^3^				
< 4500	23/73 (31.5)	1/15 (6.7)	22/58 (37.9)	.096
> 11,500	2/73 (2.7)	1/15 (6.7)	1/58 (1.7)	.024
Lymphocyte count, mm^3^				
< 1000	31/73 (42.5)	12/15 (80)	19/58 (32.8)	.001
Neutrophil count, mm^3^	3605 (2453–4714)	4723 (4095–6707)	3266 (2304–4192)	.006
< 2500	19/72 (26.4)	0	19/57 (33.3)	.013
Neutrophil/Lymphocyte ratio	2.8 (1.9–5.5)	7.0 (5.3–9.4)	2.3 (1.8–3.7)	<.001
Platelet count, mm^3^	182(136–236)	175(131–252)	186 (136–236)	.58
< 100	2/73 (2.7)	0	2/58 (3.4)	.067
C-reactive protein, mg/dL > 0.5	5/73 (75.3)	15/15 (100)	40/58 (69)	.015
Procalcitonin, ng/mL	0.05 (0.04–0.1)	0.17 (0.08–0.3)	0.05 (0.03–0.07)	.003
Ferritine, ng/mL > 300	20/25 (80)	11/11 (100)	9/14 (64.3)	.046
D-dimer, ng/mL > 500	32/68 (47.1)	10/14 (71.4)	22/54 (40.7)	.040
Prothrombin time, s	88.5 (81–99.5)	85 (73–87)	92 (85–100)	.014
Aspartate aminotransferase, U/L	27.3 (21.1–35)	26.5 (23.2–42)	27.3 (20.9–35)	.90
Alanine aminotransferase, U/L	26.1(16.4–38.6)	28.3 (15.2–38.6)	25.7 (16.4–40)	.54
Total bilirrubin, mg/dL	0.36 (0.25–0.48)	0.51 (0.38–0.7)	0.33 (0.24–0.42)	.001
Albumin, g/dL	4.05 (3.8–4.4)	3.7 (3.4–4.1)	4.1 (3.9–4.4)	.003
Lactate dehydrogenase, U/L	222 (177–296)	264 (203–411)	205 (171–275)	.011
Troponin T, ng/mL	3.2 (3–7.3)	13.3 (4–30.2)	3 (3–5.3)	.043
> 14	7/46 (15.2)	5/11 (45.5)	2/35 (5.7)	.005
Sodium, mmol/L < 135	18/77 (23.4)	11/18 (61.1)	7/59 (11.9)	<.001
Creatinine, mg/dL	0.80 (0.72–0.97)	0.87 (0.73–0.99)	0.78 (0.72–0.93)	.93

Data are median (IQR) or n/N where *N* is the total number of patients with available data

**Table 4:** Multivariate Logistic Regression Analysis Risk Factors for ICU or death for patients admitted with COVID-19

	OR (95% CI)	*p*-value
BMI	1.436 (1.042–1.979)	0.027
C-reactive protein, (mg/dL)	1.505 (1.107–2.047)	0.009

## Discussion

Chile has been one of the most affected countries by the spread of SARS-Cov-2 worldwide, and at the time of writing still struggling to contain the first wave of the Covid-19 pandemic. We report the first cohort of patients with Covid-19 in Chile and one of few in Latin America and other developing regions. The 381 patients of our cohort encompass all patients diagnosed at our institution during the first month of the pandemic and represent a significant proportion of all notified cases in Chile during the study period (*n* = 4161). The cohort mirrored the beginning of the epidemic curve (first 4 weeks), when a large proportion (~ 65%) of patients remained able to identify a risk of exposure to SARS-CoV-2; thus, many subjects attended the hospital despite having minor symptoms to receive advice and obtain a diagnosis. Importantly, in Chile, summer vacations go from December to the first week of March, so despite frontiers closing on March 16 (only 13 days after the first case), many cases were brought into the country by returning travelers. Indeed, 47 (12.3%) of patients in our cohort had recently visited what was considered at the time a high-risk country.

A high proportion of patients in our cohort had a mild presentation, with only 18 (4.7%) out of 381 requiring ICU care, consistent with prior studies during early phases of the Covid-19 outbreak [11] Interestingly, only 3 patients died during the hospitalization, representing a 3.4% of all patients requiring admission and 0.7% of the overall cohort. An in-hospital mortality rate of 3.4% is strikingly lower compared to previous large reports from Wuhan (28%), the New York Area (21%) and other European countries [12–15]. This difference could be explained by a lower threshold for admission in our cohort at a time when the healthcare system was not yet overloaded and bed capacity was high. However, an in-hospital mortality of 22% reported from Germany at a time when the healthcare capacity was not burdened argues against this as the sole explanation [14]. Also, patients in our cohort were younger (median age 49) compared to the New York, Wuhan and German cohorts (median ages 63, 56 and 72 respectively). Age has been consistently associated with disease severity and outcomes [12, 16], hence, it is likely a contributing factor in the lower mortality rates observed in our cohort. Furthermore, previous cohorts [14, 15] excluded a large proportion of patients who remained hospitalized at the time of study closure, biasing results towards an increased mortality rate due to a higher inclusion of patients who died early in the course of admission. Finally, it is important to highlight that these outcomes reflect the results of a healthcare institution serving a higher-income section of the population, and may vary from those observed in the lower resource public health system.

The cohort presented here is mostly Hispanic, an ethnic population generally underrepresented in medical research [17]. Data from the United Kingdom and USA has shown an increased risk of severe COVID-19 among ethnic minorities [18–20]. The low fatality and ICU rate of our cohort suggests that the ethnicity issue might rather be a problem of socioeconomic disparity. Studies appropriately representing minority populations are sorely needed. Indeed, failing to include an ethnically representative population leads to results that may not apply to these groups, increasing the health inequality. In Chile and other Latin American countries, future comparative studies of different socioeconomic groups will help to understand the influences of genetic factors and social inequality on the dynamics and outcomes of Covid-19.

As reported elsewhere [21], higher CRP levels were associated with increased odds of need for ICU care or in-hospital death. Further, our data suggest that elevated CRP levels in the first 24 h of admission were a biomarker for severe clinical presentation of Covid-19. CRP is an acute phase protein released mainly in response to interleukin-6 (IL-6), a cytokine that has also been associated with disease severity in Covid-19 [12]. Higher CRP levels are likely associated with a higher inflammatory response, which may correspond with increased tissue damage. CRP levels are widely available and generally cheaper than measuring IL-6 levels and therefore may represent an interesting biomarker to investigate in future studies. Increased BMI was also associated with severe disease. Several theories have been raised to explain this association, which was described before in other cohorts [22], including overactivated inflammation and immune response, decreased chest expansion, and increased expression of ACE 2, among others [22, 23]. Although the mechanisms are beyond the scope of this study, BMI seems to be a useful predictor of Covid-19 severity and therefore obese patients should be considered a high-risk population. Finally, although the SpO2/FiO_2_ index on admission was not independently associated with our main outcome (*p* = 0.051), it was very close to our pre-established level of significance. These data, along with a high biological plausibility suggests this index is worth exploring as a marker for the development of severe Covid-19 disease in future studies.

The role of other biomarkers previously identified as predictors of in-hospital mortality or ICU need were not confirmed in our cohort. These findings could be explained due to the limited number of patients included in our series along with the low frequency of occurrence of our primary outcomes (i.e. ICU admission and/or in-hospital mortality). In addition, other relevant limitations of our study include that it is a single center effort and its observational nature. Due to the latter, we did not explore treatment effects given our limited ability to appropriately correct for potential confounders. However, our data were prospectively collected with high quality standards and provide one of the few studies contributing information from developing areas of the world, in this case South America. As mentioned above, the data of this cohort mainly represent the initially affected high-income population of Chile and a time of the pandemic were the healthcare system was not yet overwhelmed. Therefore, future studies analyzing the general population attending to a wider range of hospital centers and reflecting a systemic stress created by the large number of patients infected with SARS-CoV-2 will be important to help understand the possible influence of social and health disparities, and of the system overload in the outcomes of Covid-19 patients.

## Conclusion

In conclusion, among patients in our study, SARS-CoV-2 generally caused mild illness with a case fatality rate of 0.7%. On admission, variables associated with the need of ICU care and/or in-hospital mortality included BMI and CRP, all of which are widely available in low and middle resource settings such as Latin America.

## Data Availability

The datasets used and/or analysed during the current study are available from the corresponding author on reasonable request.

## Abbreviations

BMI: Body mass index
CRP: C-reactive protein
SARS-CoV-2: Severe Acute Respiratory Syndrome Coronavirus 2
Covid-19: Coronavirus disease 19
CAS: Clínica Alemana de Santiago
ICU: Intensive care unit
CCI: Charlson Comorbidity Index
SpO_2_: Pulse oximetry saturation
FiO_2_: Fraction of inspired oxygen
IQR: Interquartile range
ACE: Angiotensin-converting enzyme
ARBs: Angiotensin II receptor blockers
